# Intraoperative Fluid Restriction is Associated with Functional Delayed Graft Function in Living Donor Kidney Transplantation: A Retrospective Cohort Analysis

**DOI:** 10.3390/jcm8101587

**Published:** 2019-10-02

**Authors:** Gertrude J Nieuwenhuijs-Moeke, Tobias M Huijink, Robert A Pol, Mostafa El Moumni, Johannes GM Burgerhof, Michel MRF Struys, Stefan P Berger

**Affiliations:** 1 Department of Anesthesiology, University of Groningen, University Medical Centre Groningen, Hanzeplein 1, 9713 GZ, Groningen, The Netherlands; m.m.r.f.struys@umcg.nl; 2 Department of Surgery, University of Groningen, University Medical Centre Groningen, Hanzeplein 1, 9713 GZ, Groningen, The Netherlands; t.m.huijink@umcg.nl (T.M.H.); r.pol@umcg.nl (R.A.P.); m.el.moumni@umcg.nl (M.E.M.); 3 Department of Epidemiology, University of Groningen, University Medical Centre Groningen, Hanzeplein 1, 9713 GZ, Groningen, The Netherlands; j.g.m.burgerhof@umcg.nl; 4 Department of Anesthesiology and Peri-operative Medicine, Ghent University, Corneel Heymanslaan 10, 9000 Gent, Belgium; 5 Department of Nephrology, University of Groningen, University Medical Centre Groningen, Groningen, The Netherlands; s.p.berger@umcg.nl

**Keywords:** fluid management, kidney transplantation, delayed graft function, goal-directed fluid therapy

## Abstract

Background: In 2016 we observed a marked increase in functional delayed graft function (fDGF) in our living donor kidney transplantation (LDKT) recipients from 8.5% in 2014 and 8.8% in 2015 to 23.0% in 2016. This increase coincided with the introduction of a goal-directed fluid therapy (GDFT) protocol in our kidney transplant recipients. Hereupon, we changed our intraoperative fluid regimen to a fixed amount of 50 mL/kg body weight (BW) and questioned whether the intraoperative fluid regimen was related to this increase in fDGF. Methods: a retrospective cohort analysis of all donors and recipients in our LDKT program between January 2014–February 2017 (*n* = 275 pairs). Results: Univariate analysis detected various risk factors for fDGF. Dialysis dependent recipients were more likely to develop fDGF compared to pre-emptively transplanted patients (*p* < 0.001). Recipients developing fDGF received less intraoperative fluid (36 (25.9–50.0) mL/kg BW vs. 47 (37.3–55.6) mL/kg BW (*p* = 0.007)). The GDFT protocol resulted in a reduction of intraoperative fluid administration on average by 850 mL in total volume and 21% in mL/kg BW compared to our old protocol (*p* < 0.001). In the unadjusted analysis, a higher intraoperative fluid volume in mL/kg BW was associated with a lower risk for the developing fDGF (OR 0.967, CI (0.941–0.993)). After adjustment for the confounders, prior dialysis and the use of intraoperative noradrenaline, the relationship of fDGF with fluid volume was still apparent (OR 0.970, CI (0.943–0.998)). Conclusion: Implementation of a GDFT protocol led to reduced intraoperative fluid administration in the LDKT recipients. This intraoperative fluid restriction was associated with the development of fDGF.

## 1. Introduction

During the procedure of organ donation and transplantation a number of potentially harmful processes will inevitably occur, affecting the viability of the kidney graft. Both donor and recipient are subjected to anesthesia and surgery, which will produce a sequence of systemic and local changes, including a significant proinflammatory and procoagulatory response [1]. The donor organ is by definition, exposed to a number of phases of injury from the moment the donor suffers from cerebral injury (in case of brain death) until the kidney is reconnected to the circulation in the recipient. These phases include a profound systemic and local proinflammatory and procoagulatory response during donor management and retrieval, associated with hypoxia and ischemia of the kidney. In addition, prolonged warm ischemia in the deceased circulatory death (DCD) donor will affect the viability of the donor kidney. These combined effects on the graft-to-be result in a cascade of renal damage that will reveal itself at the time of transplantation, when the donor kidney is reperfused in the recipient and has been named an ischemia-reperfusion injury (IRI) [2]. Typically, IRI will clinically manifest as immediate nonfunction of the transplant with the need for dialysis treatment until the graft recovers from the insult and starts eventually to function. This ‘secondary’ recovery is called delayed graft function.

DGF, a form of acute kidney injury post-transplantation, is an uncommon complication after living donor kidney transplantation (LDKT), most likely due to very short ischemia times and healthy living donors. Incidences reported vary between 1%–8% [3,4]. In transplantation with kidneys from deceased brain death (DBD) donors, however, the incidence of DGF increases to 15%–25% and may rise up to 72% in transplantation with kidneys from deceased DCD donors [5,6]. DGF is a risk factor for acute rejection (AR) and the combination of DGF and AR reduces graft and patient survival [7,8,9]. Also in the absence of AR, DGF has been shown to be an independent risk factor for long term graft loss. Reported risk factors for DGF are: deceased donor, longer ischemia times, donor and recipient older age, female donor, male recipient, history of dialysis, higher body mass index (BMI), hypertension in the donor, diabetes in the recipient, retransplantation, higher panel-reactive antibody levels, and higher human leukocyte antigens (HLA) mismatch [3,5,7,10]. This variety of risk factors underscores the complex pathological mechanisms underlying DGF. 

Regarding the intraoperative period, several studies suggest that an adequate/supranormal fluid state is associated with a reduced risk of DGF [5,7,11,12,13,14]. These studies, however, are mainly retrospective and often comprise a variety of donor types with variable incidences of DGF hampering an adequate analysis. Central venous pressure (CVP)-guided fluid therapy has been suggested until recently [11,12], but CVP does not correlate well with intravascular fluid state and its use to guide fluid therapy is currently discouraged [15]. Blood pressure and heart rate are also affected by several variables, unrelated to the circulatory state of the patient, like pain, temperature, anesthetics, and analgesics, making them less suitable as an indicator of the intravascular volume [16,17].

Recently, goal directed fluid therapy (GDFT) has been shown to improve patient outcomes after major (abdominal) surgery [18,19,20]. During 2015, our department implemented a GDFT approach in kidney transplant recipients to replace our standard intraoperative fluid regimen of four to five liters (L) of balanced crystalloids. In the first half year of 2016 a marked increase in DGF and functional (f)DGF in our LDKT population was noticed. During 2014 and 2015, respectively, 8.5% and 8.8% of the patients experienced fDGF. From January to June 2016 the incidence of functional delayed graft function (fDGF) rose to 23.0%, which was a significant increase compared to 2014 and 2015 (*p* = 0.039 and *p* = 0.021, respectively). Since the incidence of fDGF in this population has been stable over the past two decades and no protocol changes were implemented with the exception of the GDFT protocol, we questioned whether this increase in fDGF was due to the altered fluid regimen. To our surprise, a retrospective analysis revealed that the implementation of GDFT protocol had resulted in a reduced intraoperative fluid administration which seemed associated with the increase in fDGF. Based on these results, we promptly changed the intraoperative fluid protocol in September 2016 to a fixed amount of 50 mL/kg BW with a lower limit of 2500 mL and upper limit of 6000 mL (50 kg–120 kg), unless patients comorbidity determined otherwise. After six months the incidence of fDGF was back to baseline at 8.2%. 

Since we were interested in whether the amount of fluid administered intraoperatively was indeed an independent factor predicting fDGF in this LDKT population, we performed a retrospective cohort analysis of all donors and recipients in our living donor program between January 2014–February 2017.

## 2. Materials and Methods

### 2.1. Study Design and Population

This retrospective cohort analysis comprised all consecutive donor and recipient pairs of the LDKT program of the University Medical Centre of Groningen (UMCG) between January 2014 and February 2017. The Institutional Review Board approved the study (METc 201600968), which was conducted in adherence to the Declaration of Helsinki. Due to the observational and retrospective character of the analysis, the requirement for informed consent was waived. 

### 2.2. Definition of DGF

Twenty-two definitions of DGF were identified in literature based on dialysis, serum creatinine levels, urine output or a combination of these 3 [21], Most commonly used was dialysis requirement the first week after transplantation (also used in this analysis for DGF). This dialysis-based definition, however, is criticized for its subjectivity since there are center- or physician-specific thresholds for the use of dialysis after transplantation [22]. Furthermore, since approximately half of our LDKT population was transplanted preemptively, this dialysis-based definition was unsuitable for this analysis. Another definition, referred to as functional (f)DGF, is failure of serum creatinine level to decrease spontaneously by at least 10% daily on 3 consecutive days during the first postoperative week, discounting creatinine decreases due to dialysis. Moore and colleagues showed that fDGF is independently associated with reduced death-censored graft survival in contrast to DGF based on the dialysis definition and suggested a superiority of this definition over the dialysis-based definition [23]. To prevent misclassification in patients with excellent early graft function, failure of creatinine to decrease on postoperative day three was not classified as fDGF if optimal graft function had already been achieved by day 2. In this analysis, we compared patients undergoing LDKT with fDGF and without fDGF (nofDGF). 

### 2.3. Intra- and Postoperative Management and Surgical Procedure

**Figure 1:**
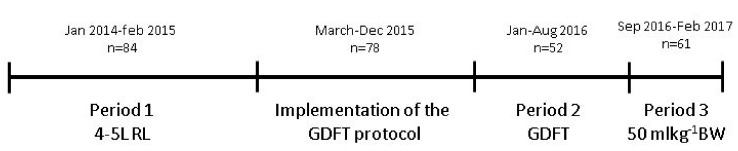
Timeline of various intraoperative fluid protocols in recipients. L: liters; RL: Ringers’ lactate; GDFT: goal directed fluid therapy, BW: body weight.

Anesthetic management was according to local protocol. Propofol was used for induction of anesthesia and either propofol or sevoflurane were used for maintenance of anesthesia. Sufentanil or remifentanil were used to control nociception and rocuronium or cis-atracurium for muscle relaxation. Until the implementation of the GDFT protocol, donors and recipients were given 4–5 L of balanced crystalloids throughout the procedure unless their comorbidity determined otherwise. During 2015, a GDFT protocol was gradually implemented in the recipients (not in the donors). For a detailed description of this protocol, see below. From September 2016 fluid protocol in recipients was changed to a fixed amount of 50 mL/kg BW intraoperatively. Timeline of fluid management in recipients is given in Figure 1. Fluid management in donors was not actively changed during our observation period. Regarding the type of fluid, predominantly Ringers’ lactate (RL) was used. If hyponatremia occurred RL was replaced by 0.9% saline. Colloids were not given and administration of blood products was according to our local transfusion protocol with thresholds based upon patients comorbidity. Regarding hemodynamics, the goal was to keep the blood pressure within 80% range of the baseline blood pressure of the patient. As baseline, we used blood pressure measured at the preoperative visit. If hypotension occurred, the first step was to adjust depth of anesthesia or analgesia. If that was insufficient or not possible, patients received one or more doses of ephedrine or phenylephrine or a continuous infusion of noradrenaline was started. Kidney donation was performed using a hand-assisted laparoscopic approach. Thereafter the kidney was flushed and perfused with cold University of Wisconsin solution (ViaSpan®, BMS, Bruxelles, Belgium or CoStorSol®, Bridge to Life, Elkhorn, WI, USA) and stored on ice. Transplantation was performed according to local, standardized protocol. Postoperative fluid management comprised 1 L NaCl 0.45%-Glucose 2.5% per 24 h, complemented with the volume of diuresis in the former hour. 

### 2.4. Goal-Directed Fluid Therapy Protocol.

GDFT was performed with the use of the FloTrac® in combination with the EV1000® monitor (Edwards Lifesciences Corporation, Irvine, CA, USA). The system was used according to manufacturer’s instructions. A standard institutional GDFT protocol was used with adjustment of the goal. Instead of a stroke volume variation (SVV) < 12%, commonly used in abdominal surgery, we aimed for a SVV < 10% throughout the procedure. When the SVV was >10% additional fluid was given until SVV was <10%. If SVV < 10%, fluid administration was left to the discretion of the attending anesthesiologist, however, when cardiac index (CI) was below age-adjusted normal values, a noradrenaline infusion was started. If measurement of the SVV was not possible (e.g., due to cardiac arrhythmias) a protocol based on stroke volume (SV) was used. In this case, if a fluid bolus of 250 mL resulted in an increase of the SV of 10%, additional fluid was given, if not, the trend of the SV was monitored and fluid administration was left to the discretion of the attending anesthesiologist. When SV decreased >10%, additional fluid was given. The FloTrac® was used with the EV1000 monitor, which does not communicate with our digital PDMS. Therefore SV, SVV, and CI values could not be retrieved for this analysis.

### 2.5. Patient Data

Demographic and postoperative data were obtained from digital patient medical records. The following variables were taken into account: age, gender, BMI, smoking, hypertension, use of antihypertensive drugs, measured glomerular filtration rate (mGFR) with use of iodine 125-iothalamate in the donor, blood pressure (measured the day of hospital admission), difference in blood pressure between donor and recipient measured by systolic/diastolic/mean of the recipient minus systolic/diastolic/mean of the donor, underlying kidney disease, number of HLA mismatches, history of dialysis, related or unrelated donor transplantation. For all recipients, the age-adjusted Charlson comorbidity index (CCI) [24] and length of hospital stay was calculated. Intraoperative data were retrieved from our digital patient data monitoring system (PDMS, CS-EZIS, Chipsoft B.V., Amsterdam, the Netherlands) and consisted of duration of surgery, intraoperative volume and type of fluid, cumulative hypotensive periods defined as a systolic blood pressure < 80 mmHg and MAP < 60 mmHg, intraoperative use of vasoactive substances, ischemia times, left/right kidney, side of implantation, number of arteries, sacrifice of an accessory artery, and urinary output the first 2 h postoperatively. Regarding the use of vasoactive substances, patients were scored on receiving one or more boluses of ephedrine and/or phenylephrine and whether or not noradrenaline was administered as a continuous infusion. Additionally, the maximum noradrenaline infusion rate during the procedure was noted. This was grouped into 3 categories: low infusion rate (0.02–0.10 mg/h), intermediate (0.10–0.20 mg/h), and high (>0.20 mg/h) infusion rate. 

### 2.6. Statistics

For the statistical analysis SPSS version 23 (IBM Corp, Armonk, NY, USA) and GraphPad Prism version 7.02 (GraphPad Software Inc, La Jolla, CA, USA) were used. We performed univariate analyses to identify factors associated with fDGF. Categorical data were analyzed by chi-square or Fisher’s exact tests. Continuous data were analyzed with an unpaired t-test in the case of normally distributed values. If variables were not normally distributed Mann–Whitney test was applied. Multivariate analysis was performed by means of binary logistic regression. We adjusted the amount of fluid administered intraoperatively in recipients for potentially relevant confounders with high significance in the univariate analysis. Additionally, we were interested in the impact of implementation of our GDFT protocol on the incidence of fDGF and on the amount of fluid administered intraoperatively. We therefore analyzed these data between the different time periods 1–3 (described above) with the use of Fisher’s exact test and Kruskal–Wallis test. Post-hoc analysis with Mann–Whitney was used. Values are given as number (%), mean ± standard deviation (SD) or median with interquartile range (IQR). All reported *p*-values are two-sided. A *p*-value of 0.05 or less was considered significant.

## 3. Results

### 3.1. Univariate Analysis 

#### 3.1.1. Patient Characteristics

Between January 2014 and February 2017, 275 living donor kidney transplant procedures were performed in our center. Of the 275 recipients, 31 patients experienced fDGF and 244 recipients did not (nofDGF). Donor and recipients characteristics of fDGF and nofDGF kidneys are listed in Table 1. There were no statistically significant differences in baseline characteristics and kidney function (mGFR) in donors of kidneys with our without fDGF. Recipients developing fDGF were more likely to be dialysis-dependent at the time of transplantation (25 (81%) vs. 105 (43%), *p* < 0.001). The composition of the group of dialysis dependent patients did not differ between nofDGF and fDGF recipients. In the nofDGF group 76 (72%) patients were on hemodialysis at the time of transplantation and 29 (28%) on peritoneal dialysis. In the fDGF group, this was the case for 19 (76%) and six (24%), respectively. All patients on hemodialysis were dialyzed the day before transplantation to 1 kg above dry weight.

#### 3.1.2. Intra- and Postoperative Data 

Intraoperative data of donors of fDGF and nofDGF kidneys showed no differences with exception of the total amount of fluid, in which donors of fDGF kidneys received less fluid intraoperatively, which was the case for total volume (3545 mL (778.2) vs. 3845 mL (799.1), *p* = 0.050) and mL/kg BW (45 mL/kg BW (10.3) vs. 49 mL/kg BW (11.4), *p* = 0.053). 

Recipients who developed fDGF received significantly less intraoperative fluid, which was the case for the total amount of fluid (3000 mL (2250–3680) vs. 3500 mL (2900–4075), *p* = 0.023) and mL kg-1BW (36 mL/kg BW (25.9–50.0) vs. 47 mL/kg BW (37.3–55.6), *p* = 0.007). Predominantly RL was given, but in case of hyponatremia RL was partially replaced by saline. This was the case in 48 (20%) of the recipients without fDGF and in 8 (26%) of the patients with fDGF (*p* = 0.477). Median volume replaced by saline was 1000 mL (500–2000) in the nofDGF group and 800 mL (500–1075) in the fDGF group (*p* = 0.865). Blood loss was comparable between groups and transfusion of red blood cells was applied in 10 (4.1%) of the patients in the noFDGF group and two (6.4%) of the fDGF group. Patients showed no difference in hypotensive periods, but recipients experiencing fDGF were treated more frequently with noradrenaline continuous infusion (*p* = 0.034), which was only the case for low dose infusion with a maximum of 0.1 mg/h. For noradrenaline administered at higher dosage (>0.1 mg/h), there was no difference between the two groups. fDGF was associated with a lower urine output during the first two hours after transplantation (*p* = 0.005 for the first hour and *p* = 0.002 for the second hour). Ten patients in the fDGF group were dialyzed after transplantation versus zero patients in the nofDGF group (*p* < 0.001). Eight of these kidneys gained function after a mean of 10.3 (3.1) days. Two kidneys suffered primary nonfunction due to a combination of ATN and mild antibody-mediated rejection (patient 114, transplanted June 2015) and non-HLA-mediated hyperacute rejection (patient 273, transplanted November 2016). Recipients experiencing fDGF showed a longer hospital stay (14 (10–20) vs. 9 (7–13) days *p* < 0.001) (Table 2). 

### 3.2. Multivariate Logistic Regression Analysis

In the unadjusted analysis, a higher intraoperative administered fluid volume was associated with 3% lower odds for the development of fDGF per mL/kg BW (OR 0.967, CI (0.941–0.993), model 1). We adjusted for potentially relevant confounders with high significance in the univariate analysis, i.e., a history of dialysis and the use of intraoperative noradrenaline, after which the relationship was still apparent (OR 0.970, CI (0.943–0.998), model 2). Since the intraoperative amount of fluid in the donors approached significance in the univariate analysis with lower volumes given in the fDGF group, we also adjusted for amount of fluid in the donor, after which the relationship was still apparent (OR 0.969, CI (0.941–0.997), model 3) (Table 3).

### 3.3. Influence of the GDFT Protocol on the Intraoperative Fluid Volume. 

Additionally, we were interested in the impact of implementation of our GDFT protocol on the incidence of fDGF and on the amount of fluid administered intraoperatively. The GDFT protocol was gradually implemented during 2015 and in 2016 (up to September) all recipients were treated following this protocol (Figure 1). Data of the EV1000 monitor were not recorded in our PDMS, therefore we were unable to see which patients in 2015 were treated according the GDFT protocol and disregarded this period (March 2015–December 2015) in this specific analysis. We compared patients transplanted between January 2014–February 2015 (period 1, *n* = 84, old protocol) to patients transplanted between January 2016–June 2016 (period 2, *n* = 52, GDFT protocol) and patients transplanted between September 2016–February 2017 (period 3, *n* = 61, new protocol).

**Figure 2:**
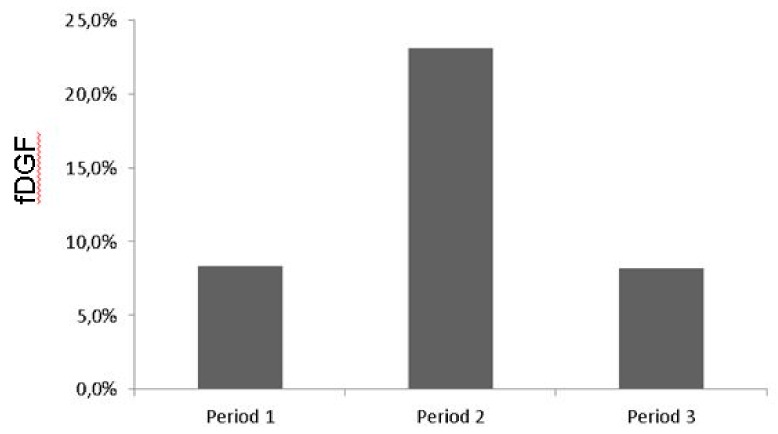
Incidence of fDGF in recipients during the different time periods. Period 1: January 2014–February 2015, old protocol, 4–5 L RL. Period 2: January–June 2016, GDFT protocol. Period 3: September 2016–February 2017, new protocol, 50 mL/kg BW. *p* = 0.029.

Incidence of fDGF during the different periods are shown in Figure 2. Implementation of GDFT was accompanied by an increase in fDGF from 8.3% in period 1 to 23% in period 2. The implementation of the new protocol in period 3 resulted in a reduction of the incidence of fDGF back to baseline (8.2%, *p* = 0.029).

**Figure 3:**
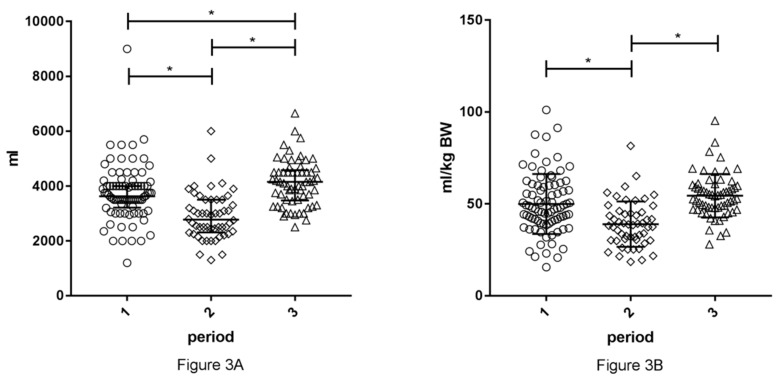
Volume of fluid administered intraoperatively in recipients during the different time periods. Period 1: January 2014–February 2015, old protocol, 4–5 L RL. Period 2: January–June 2016, GDFT protocol. Period 3: September 2016–February 2017, new protocol, 50 mL/kg BW. Volumes are given in mL (**A**) and mL/kg BW (**B**).

Total amount of intraoperative administered fluid and mL/kg BW in recipients in the different time periods are shown in Figure 3A,B, respectively. Total amount of fluid and mL/kg BW were significantly different between the three time periods (*p* < 0.001, *p* < 0.001). Implementation of the GDFT (period 2) resulted in a decrease of intraoperative fluid administration compared to our old protocol (period 1), which was the case for total volume (2775 mL (2313–3500) vs. 3625 mL (3213–4000), *p* < 0.001) and mL/kg BW (38 mL/kg BW (30.3–45.3) vs. 48 mL/kg BW (40–60), *p* < 0.001). The implementation of the new protocol (period 3) resulted in an increase in intraoperative fluid administration to 4150 mL (3475–4575) mL and 54 mL/kg BW (47.4–60.1) compared to the old (total volume *p* = 0.037, mL/kg BW *p* = 0.053) and GDFT (total volume *p* < 0.001, mL/kg BW *p* < 0.001). 

## 4. Discussion

This retrospective cohort analysis study shows that intraoperative fluid restriction in recipients is associated with fDGF in living donor kidney transplantation. Additionally, we showed that the implementation of a GDFT with a goal set at SVV < 10% led to a reduction of intraoperative fluid administration, on average by 850 mL in total and 21% in mL/kg BW, compared to our old protocol of 4–5 L of RL. In our opinion, this analysis provides valuable information for other centers when changes in intraoperative fluid management during kidney transplantation are considered.

Four to five liters of RL was the standard intraoperative fluid protocol in kidney transplantation in our center for over 15 years. This may seem rather liberal, but problems due to hypervolemia were rarely seen. However, following new trends on GDFT [24], a personalized intraoperative fluid approach seemed more appropriate in this group of patients presenting with a variety of fluid states at the time of surgery. Therefore, when in 2015 an intraoperative GDFT protocol was introduced in our center for several surgical procedures, we included the kidney transplant program in this implementation. Since there is no evidence in current literature on what goal to aim for, we adjusted the standard institutional GDFT protocol of SVV < 12%, commonly used in abdominal surgery, to a more generous goal in fluid administration of SVV < 10%. The implementation of this protocol resulted in a reduction in the amount of fluid administered intraoperatively in contrast to previous studies comparing GDFT to a “standard” protocol, which generally reported an increase of the amount of fluid. This could be due to the fact that most of these studies compare GDFT with a rather restrictive fluid protocol, which was general practice before GDFT was introduced. Kidney transplantation, however, has always been an exception on this restrictive trend and most centers use a rather liberal fluid protocol during this procedure. Another factor could be the performance of the FloTrac®-system in predicting fluid responsiveness in this specific patient category. GDFT and the performance of the FloTrac®-system has predominantly been validated in cardiac and abdominal surgery, liver transplantation, and septic patients. Patients with end-stage renal disease (ESRD) and especially patients on HD develop morphologic and functional cardiovascular changes. They often present with severe arterio- and atherosclerosis, inducing arterial stiffening and systolic or diastolic dysfunction. Since SVV is calculated as the percentage change of SV to the mean, derived from an arterial pulse contour analysis, it is conceivable that these cardiovascular changes influence the performance of the FloTrac®-system in predicting patients fluid state. Only one pilot study presents the effect of fluid loading on SVV measured with the use of the FloTrac®-system in patients with ESRD on HD. In this study, HD patients undergoing vascular surgery presented with a broad range of SVV (16.2 ± 6.0) after induction of anesthesia. After a fluid bolus of only 500 mL of a colloid solution almost all patients showed a SVV < 10% (6.2 ± 2.8), the threshold in our protocol [25].

The debate on perioperative fluid management is still ongoing. Controversy exists regarding assessment of the intravascular volume state, which goals to aim for, how to measure these goals, and what type of fluid should be used. Hypovolemia leads to a decreased oxygen supply to organs and tissues and may cause hypoxia, which can lead to organ dysfunction. Hypervolemia, on the other hand, can damage the endothelial glycocalyx resulting in a fluid shift from the intravascular compartment to the interstitial space and tissue edema [26]. Shin and colleagues report in their large cohort analysis of 92.094 patients undergoing noncardiac surgery that both too little and too much intraoperative fluid is associated with increased morbidity, mortality, costs, and length of hospital stay [27]. Myles and colleagues randomLy assigned 3000 patients undergoing a major abdominal procedure to a restrictive or liberal fluid regimen. In their study, a restrictive regimen was associated with increased risk of acute kidney injury with a hazard ratio of 1.71 (95% CI 1.29–2.27) [28]. These studies, however, do not take kidney transplant recipients into account. In the normal kidney, blood flow is regulated by an autoregulatory mechanism, ensuring adequate perfusion in a broad blood pressure range by afferent and efferent arterioles. In the transplanted, denervated kidney, this haemodynamic autoregulation is impaired making the renal blood flow linearly dependent on the systemic blood flow [29,30,31]. Furthermore, reperfusion of the ischemic kidney can be followed by vasoconstriction in the afferent arterioles. This may result in a reduced GFR due to a decrease in glomerular transcapillary hydraulic pressure difference [7,32,33]. Ensuring an adequate volume state in this specific patient category, therefore, is essential to obtain an adequate circulation both on macro- and microcirculatory level. Recently, Cavalari and colleagues reported the results of their prospective observational study, in which they compared a prospectively observed cohort of 33 deceased donor kidney transplant recipients treated with a GDFT protocol to a historical cohort of 33 kidney transplant recipients treated with their conventional fluid therapy [34]. They observed a significant reduction of cardiovascular complications, DGF. and surgical complications in the GDFT group. Surprisingly, in this study both groups received the same amount of fluid throughout the transplant procedure. Studies including deceased donor kidneys, however, comprise a variety of donor types with variable incidences of DGF hampering an adequate analysis and conclusions.

The most important predictor of fDGF in our analysis was dialysis dependency at the time of transplantation. A history of dialysis and especially hemodialysis prior to transplantation is a known risk factor of DGF [5,7,35,36]. Hypovolemia at the time of transplantation is one of the proposed underlying mechanisms [37]. Our hypothesis before implementation of the GDFT protocol was that these hypovolemic dialysis patients would present with higher SVV at time of surgery, demanding more fluid intraoperatively, compared to the relatively normovolemic or slightly hypervolemic preemptively transplanted patients. Surprisingly, comparable amounts of fluids were given to the two groups. 

In our GDFT protocol, noradrenaline was used when CI was below an age-adjusted value. Therefore, an increased use of noradrenaline was seen in period 2 compared to period 1 (71% vs. 41% *p* = 0.001) due to the implementation of the GDFT. In period 3, the use of noradrenaline decreased to 50% of the patients. In the univariate analysis, the use of noradrenaline was correlated with development of fDGF, but after multivariate logistic regression this was no longer the case. However, Morita and coworkers showed that in a rat model, transplanted kidneys responded to sympaticomimetics with a reduction in renal blood flow (RBF) in contrast to the increase in RBF seen in native rat kidneys [38].

There are some limitations of this analysis that have to be addressed: A major limitation is that we were unable to evaluate outcome directly according to the fluid protocol (4–5L RL vs. GDFT) and are unable to present information or draw any conclusions regarding actual SV, SVV, CO or CI values and their relation to the observed increase of fDGF. Other limitations are those of a retrospective observational trial. There is the potential of confounding by unmeasured factors. Regarding postoperative fluid volume, the exact amount of fluid given could not be retrieved in a reliable way from our PDMS and is therefore not implemented in this analysis. Postoperative fluid management was according to a standardized protocol and comprised of 1 L NaCl 0.45%-Glucose 2.5% per 24 h, complemented with the volume of diuresis in the former hour. This means that when the kidney produces less urine the patient will be given less fluid postoperatively. Since fDGF was associated with a lower urinary output the first two hours, it is very likely that patients experiencing fDGF received less fluid postoperatively. Whether this contributed to development of fDGF or is more of a symptom remains unknown. Backpressure from congested tubules obstructed with cellular debris may contribute to a reduction in GFR [39,40]. A higher volume of urine in the first hours may have led to washout of this debris.

Finally, due to the fact that there are only 31 events there is always the possibility of overestimating the strength of associations using a multivariate analysis. A strong argument, however, is that no policy changes were implemented during the study period with the exception of the intraoperative fluid regimen. Furthermore the incidence of fDGF in our LDKT population has been stable over many years and after changing the fluid regimen back to a more liberal fixed amount of 50 mL/kg BW the incidence of fDGF instantly returned to baseline.

DGF after transplantation is a clinically relevant problem. It is associated with an increase in morbidity, patient anxiety, increased risk of acute rejection, and additional diagnostic procedures and costs. In our population the median hospital stay in patients experiencing fDGF was prolonged by five days. Furthermore, this study shows that strict protocols for perioperative fluid management are needed when studies in kidney transplantation are designed. Fluid restriction can be an important risk factor for DGF, a frequently used primary end point, even in the setting of LDKT.

## 5. Conclusions

Implementation of a goal-directed approach to fluid administration with a goal set at a SVV < 10% throughout the procedure led to reduced intraoperative fluid administration in the LDKT recipients in our center. This intraoperative fluid restriction was associated with the development of more fDGF. A thorough validation of GDFT protocols in patients with renal insufficiency is warranted before these are implemented in this population.

## Figures and Tables

**Table 1:** Donor and recipient demographics. Data given as number (%), mean (SD), or median (IQR).

	nofDGF	fDGF	*p*
**Donor**	***N = 244***	***N = 31***	
Age year	54 (11.6)	51 (12.4)	0.104
Gender male	117 (48%)	20 (65%)	0.089
BMI	26.1 (3.0)	25.1 (2.7)	0.075
Smoking	67 (27%)	13 (42%)	0.140
Blood pressure			
S-RR mmHg	136 (15.3)	136 (11.8)	0.848
D-RR mmHg	79 (73–84)	81 (73–86)	0.548
MAP mmHg	98 (9.4)	98 (6.7)	0.897
Hypertension	38 (16%)	2 (6%)	0.277
Anti-hypertensive drugs			
Diuretics	11	1	>0.999
Β-blocker	13	1	>0.999
Ca antagonist	10	0	0.610
ACE-I	4	0	>0.999
AT-II-ant.	16	1	0.703
mGFR			
Non-stimulated mL/min	109 (97–23)	107 (95–128)	0.846
Stimulated mL/min	116 (103–133)	118 (100–140)	0.764
∆GFR	7 (2–12)	7 (−1–12)	0.810
**Recipient**	***N = 244***	***N = 31***	
Age year	54 (41−61)	55 (43−62)	0.991
Gender male	138 (57%)	21 (68%)	0.254
BMI	25.6 (22.6–28.4)	25.8 (24.0–29.8)	0.267
Smoking	45 (18%)	7 (23%)	0.626
Blood pressure			
S-RR mmHg	143 (20.4)	138 (23.7)	0.196
D-RR mmHg	79 (73–84)	81 (73–86)	0.548
MAP mmHg	97 (9.4)	98 (6.6)	0.897
∆ blood pressure with donor			
∆ S-RR mmHg	7.1 (22.8)	2.5 (29.1)	0.308
∆ D-RR mmHg	3.1 (13.9)	1.0 (15.0)	0.336
∆ MAP mmHg	4 (−6–14)	8 (−10–12)	0.756
Hypertension	175 (72%)	21 (68%)	0.675
Antihypertensive drugs			
Diuretics	84 (34%)	8 (25%)	0.421
Β-blocker	124 (51%)	10 (32%)	0.058
Ca antagonist	131 (54%)	15 (48%)	0.703
ACE-I.	46 (19%)	5 (16%)	0.811
AT-II-ant	55 (23%)	7 (23%)	>0.999
CCI	3 (2–4)	3 (2–6)	0.157
Underlying kidney disease			
DM	15 (6%)	5 (16%)	0.358
PKD	57 (23%)	5 (16%)	0.495
Systemic autoimmune diseases	25 (10%)	3 (10%)	>0.999
Glomerulonephritis	47 (19%)	4 (13%)	0.4713
Other	100 (41%)	14 (45%)	0.701
HLA mm < 3	55 (23%)	8 (25%)	0.655
Dialysis dependent	105 (43%)	25 (81%)	<0.001 *
LURD	164 (67%)	19 (61%)	0.547

fDGF: functional delayed graft function; BMI: body mass index; S-RR: systolic blood pressure; D-RR: diastolic blood pressure; MAP: mean arterial pressure; ACE-I: angiotensin-converting enzyme inhibitor; AT-II-ant: angiotensin II receptor antagonist; CCI: Charlson comorbidity index; mGFR: measured glomerular filtration rate measured with use of iodine 125-iothalamate; DM: diabetes mellitus; PKD: polycystic kidney disease; HLA: human leucocyte antigen; LURD: living unrelated donation; *: statistically significant.

**Table 2:** Intra- and postoperative donor and recipient data. Data given as number (%), mean (SD), or median (IQR).

	nofDGF	fDGF	*p*
**Donor**	***n = 244***	***n = 31***	
Duration min	227 (38.2)	216 (36.8)	0.134
Fluid			
Total mL	3845 (799.1)	3545 (778.2)	0.050*
mL/kg BW	49 (11.4)	45 (10.3)	0.053
Intraoperative blood pressure			
S-RR ≤ 80 mmHG	137 (56%)	21 (68%)	0.251
Cumulative duration (min)	10 (5–15)	10 (5–15)	0.772
Vasoactive substances			
Ephedrine	178 (73%)	25 (71%)	0.515
Phenylephrine	22 (9%)	4 (13%)	0.512
Noradrenaline	61 (25%)	11 (35%)	0.277
**Recipient**	***n* = 244**	***n = 31***	
Duration min	212 (189–239)	224 (190–260)	0.390
Fluid			
Total mL	3500 (2900–4075)	3000 (2250–3680)	0.023*
mL/kg BW	47 (37.3–55.6)	36 (25.9–50.0)	0.007*
Intraoperative blood pressure			
S-RR < 80 mmHg	49 (20%)	6 (19%)	>0.999
Cumulative duration min	5 (5–10)	7.5 (4.5–11.2)	0.679
MAP < 60 mmHg	93 (38%)	11 (35%)	0.846
Cumulative duration min	10 (5–10)	5 (5–20)	0.759
Vasoactive substances			
Ephedrine	93 (38%)	16 (52%)	0.174
Phenylephrine	26 (11%)	3 (10%)	>0999
Noradrenaline	129 (53%)	23 (74%)	0.034*
0.02–0.10 mg h^−1^	37 (15%)	10 (32%)	0.024*
0.10–0.20 mg h^−1^	42 (17%)	7 (23%)	0.459
>0.20 mg h^−1^	49 (20%)	6 (19%)	>0.999
Ischemia times (min)			
WIT	3 (3–4)	3 (3–4)	0.724
CIT	154 (140–173)	158 (141–178)	0.646
WIT2	39 (33–45)	38 (33–45)	0.982
Kidney left	177 (73%)	19 (61%)	0.209
Right fossa	203 (83%)	26 (84%)	>0.999
>1 artery	49 (20%)	8 (26%)	0.482
Artery sacrificed	11 (5%)	4 (13%)	0.074
Blood loss (mL)	250 (150–400)	250 (162.5–500)	0.499
Urineproduction	***n* = 230**	***n = 30***	
1st h (mL)	405 (250–675)	255 (75–512)	0.005*
2nd h (mL)	350 (250–550)	183 (64–462)	0.002*
	**n = 244**	***n = 31***	
Dialysis after transplantation	0 (0%)	10 (32%)	<0.001*
Length of hospital stay days	9 (7–13)	14 (10–20)	<0.001*

Min: minutes; BW: bodyweight; S-RR: systolic blood pressure; MAP: mean arterial pressure; WIT: warm ischemia time; CIT: cold ischemia time: WIT2: warm ischemia time 2; *: statistically significant

**Table 3:** Multivariate logistic regression on risk factors of functional delayed graft function (fDGF).

Model	Odds ratio (95% CI)	*p*
**1. Unadjusted analysis, model 1**		
Amount of fluid administered intraoperatively, recipient, mL/kg BW	0.967 (0.941–0.993)	0.015
**2. Adjusted analysis, model 2**		
Amount of fluid administered intraoperatively, recipient, mL/kg BW	0.970 (0.943–0.998)	0.036
No dialysis dependence at time of transplantation	0.186 (0.073–0.475)	<0.001
Use of noradrenaline continuous infusion yes/no	2.018 (0.834–4.878)	0.119
**3. Adjusted analysis, model 3**		
Amount of fluid administered intraoperatively, recipient, mL/kg BW	0.969 (0.941–0.997)	0.029
No dialysis dependence at time of transplantation	0.181 (0.071–0.464)	<0.001
Amount of fluid administered intraoperatively, donor, mL/kg BW	0.978 (0.942–1.014)	0.231
